# Predictor Variables in the Spread of Chagas Disease in Rural Areas

**DOI:** 10.3390/pathogens13050394

**Published:** 2024-05-08

**Authors:** Liziana de Sousa Leite, Valéria Christina de Rezende Feres, Paulo Sérgio Scalize

**Affiliations:** 1 Post-Graduation Program in Environmental Sciences (CIAMB), Federal University of Goiás, Goiania 74605-170, Brazil; lizianaleite@gmail.com; 2 Faculty of Pharmacy, Federal University of Goiás, Goiania 74605-170, Brazil; valeriacris@ufg.br; 3 Post-Graduation Program in Environmental Sciences (CIAMB) and the Post-Graduation Program in Sanitary and Environmental Engineering (PPGEAS), Federal University of Goiás, Goiania 74605-170, Brazil

**Keywords:** neglected tropical disease, risk factors, dissemination, trypanosomiasis, epidemiologic variables, sanitation

## Abstract

Over a hundred years ago after the discovery of Chagas disease (CD) in Brazil, the World Health Organization estimates a number of 6 to 7 million people infected by *Trypanosoma cruzi* worldwide. Therefore, the goal of this work was to identify variables related to the spread of infection by *T. cruzi* in humans living in rural areas, seeking predictor variables. A systematic review of the literature has been conducted, with a search in the Scopus platform, using the search string “Chagas disease” and “rural”, resulting in 85 valid and analyzed scientific studies (1977 and 2022). Twenty-seven predictor variables have been acquired, and 19 of them have been grouped, such as: socioeconomic and educational, housing, environmental, sanitary, and cultural; and 8 variables related to *T. cruzi* seropositive individuals. The predictor variables yielded significant results (*p*-value < 0.05) in 59.5% of the cases (195/328), with a median of 66.7%. In other words, studies relating to 50% of the 27 variables showed significance equal to or greater than 66.7% of the time. The independent variables with the highest proportion of significant data (*p*-value < 0.05) were Education (87.6%), Intradomicile building (70%), Domestic animals (69.6%), and Triatomines (69.2%) in the households. Some variables reached 100%; however, few articles were found, indicating the need for further research, especially for Sanitation and Culture. It has been concluded that, in the several contexts found, the social vulnerability and lack of information led the individual to living in environments where inhabitability is inadequate, to perform limited work activity and develop habits and behaviors which impair them in an environmental insalubrity situation, favorable to the access of vectors and pathogens of anthropozoonoses such as CD.

## 1. Introduction

Chagas disease (CD) was discovered in 1909 in Brazil, when its vector (triatomine), the pathogen (*Trypanosoma cruzi*) and the infection in humans have been identified and associated to inhabitants of wattle houses in the rural environment [1], propitious to the vector proliferation and spread of *T. cruzi* [2,3].

*Trypanosoma cruzi* is a protozoan found in the intestine and Malpighian tubule of the infected invertebrate (triatomine) and in the contaminated vertebrate animal (mammal) bloodstream and tissues, and it compromises, in human beings, the cardiac and digestive functions by parasitic sheltering in cells [4,5]. In the transmission cycle, the triatomine becomes a vector after feeding on mammalian blood contaminated by *T. cruzi* [5]. Within its intestine, the protozoan multiplies (epimastigote form) and assumes the infective flagellated trypomastigote form [4]. Due to the insect’s habit of defecating and/or urinating after biting, it transmits the protozoan *T. cruzi* to humans or other vertebrate mammals. This occurs when the skin is scratched, introducing the invertebrate’s feces into the vertebrate’s bloodstream at the site of the bite [4,5].

The CD vector, arthropod of the Triatominae (Hemiptera, Reduviidae) subfamily, is also known as the kissing bug, conenose bug or vampire bug. In Brazil, it is referred to as “barbeiro, chupão, procotó, bicudo or chipó”, while in Hispanic countries, it goes by “barber, chuoetón, protocó bicudo or chipos”. This hematophagous insect exhibits nocturnal behavior [5,6,7] and is predominantly found in South and Central Americas. Some species also inhabit regions of the of the USA, center-southern Africa, southeastern Asia and northern Australia [5,6,8]. However, CD is found in the north of the American continent [9,10], the European continent [11], the East [12] and in 21 Latin American countries [3,13,14]. Still, out of the boundaries of Latin America, it is possible to notice a lack of information by health professionals regarding the need to investigate CD, especially in patients which emigrate from endemic regions, influencing spread, diagnosis and treatment of the disease [11,15,16]. As it is asymptomatic in most hosts in which the disease evolved from the acute to chronic stage, without precocious diagnostics, years or decades after infection, 30% of the affected begin feeling cardiac symptoms, and 10% feel symptoms in the digestive tract [5,17,18].

Other forms of transmission include transplacental transmission and, more rarely, through blood transfusion or organ transplantations, laboratory accidents, and sexual transmission. Additionally, transmission can occur orally, mainly the ingestion of food contaminated with *T. cruzi*, which is related to the acute Chagas disease (ACD) [5,19].

In the US, the autochthonous transmission form is considered rare, even with the existence of 10 triatomine specimens in the wild environment which are able to host the *T. cruzi*. There is also the possibility the risk of infection through blood transfusion and oral ingestion of contaminated food by the anal glands of skunks or the consumption of raw beverages [10,15,20,21]. In Mexico, studies on triatomine infections [22,23] confirmed the risk of *T. cruzi* infection when encountering infected dogs [24] and humans [9].

In Central America, CD is considered endemic in seven countries, with an estimate of 12% of the population under risk of infection [25,26]. In Spain and Japan, the presence of CD is considered to derive from the immigration of Latino-Americans, such as Brazilians, Colombians and Bolivians, and of congenital transmission [11,16,27] being highlighted the difficulty of access to health services by immigrants in Japan [12].

In Latino-American countries, considered endemic for CD, there are still places with high seroprevalence, such as in the province of Cordillera, located in the Bolivian Chaco, where seroprevalence of 22.0% in children with average age of 13 years old has been found [28]. Among many forms of CD spread in these countries, we have: vector transmission, such as in Brazil [29]; congenital transmission, such as in Ecuador [30] and Colombia [31]; oral transmission, as noticed in a Venezuelan school [32]; and by blood transfusion or organ transplant, such as in Argentina [33].

Over a hundred years after the discovery of CD, the World Health Organization (WHO) [3] estimates an approximate number of 6 to 7 million of people infected by *T. cruzi* throughout the world [13,15]. Immigrates from endemic countries and migratory flows played a role its expansion in nonendemic countries [12,34]. In the Americas, 30.000 new Chagas cases and 10.000 deaths are related every year, with an estimated 75 million people under risk of contracting CD [35]. In Brazil, a Latino-American country where CD was discovered, in the last 15 years there were several reported cases of ACD related to oral transmission, especially in the Amazon region. This transmission occurs through the consumption of raw foods contaminated with the feces of the vector infected by *T. cruzi*, as well as through extra-domiciliary vectorial transmission resulting from accidental encounters with the insect in the wild environment [36,37]. However, as in other Latin American countries, due to deforestation and proximity to the houses of the wilderness ecotope, different vector specimens have been adapting to artificial environments, such as intra- and peridomiciles [3,14,36,38,39]. In these environments, these vectors find sustenance not only from humans and wildlife, but also domestic and synanthropic animals, such as dogs, chickens, marsupials and rodents [39,40,41].

Dr. Chagas, the medical researcher who discovered CD, in his studies regarding American trypanosomiasis, has noticed characteristics common to his patients: poor, rural, marginalized, urban physicians and academics. In 1949, Dr. Mazza added to the disease cycle’s characteristics the poor hygienic and socioeconomic characteristics [42]. This leads to the access to public services, such as sanitation, that is, potable water supplying, sanitary sewer system, rainwater and solid waste management [43], living in a household built with good quality building materials, giving it the status of inhabitable, within the concept of rural inhabitability [44].

The socioeconomic and cultural factors associated with CD, exacerbated by poverty [26,34,45], not only increase the risk of disease transmission in urban and periurban areas [46,47], but also contribute to its spread in rural populations. This is primarily because rural dwellings are typically constructed with adobe, bahareque, or plant materials, which are conducive to triatomine infestation [3,26]. Additionally, customary practices such as stockpiling materials and establishing animal enclosures in peridomiciliary areas further elevate the risk. Moreover, the proximity of rural households to wilderness environments, where activities like agriculture, cattle farming, and extractivism are common, facilitates contact with the vector [13,38,44,45,48,49].

It Is essential to know the risk variables of CD spread and observe its forms of transmission, to base strategies for its control and prevention. This work aimed to identify predictor variables that explain the spread of *T. cruzi* infection in humans in rural areas.

## 2. Materials and Methods

**Figure 1:**
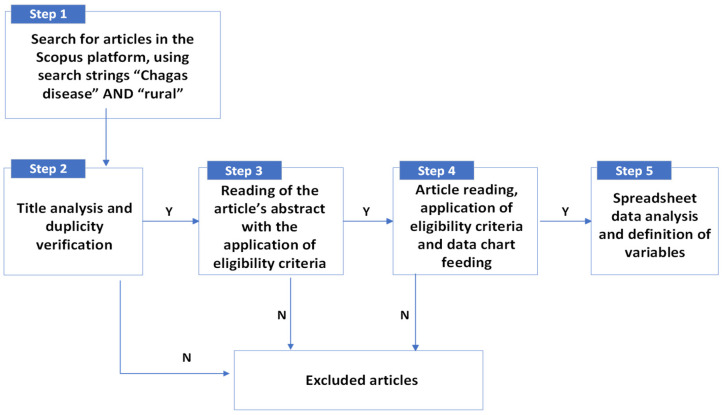
Flowchart of the development process of the systematic review of the literature. Note: Y = pertinent to the subject; N = not pertinent to the subject.

The bibliometric analysis deciphers and maps the cumulative scientific knowledge and the nuances of field works, giving meaning to nonstructured data [50]. For the development of this research, a systematic review of the literature (SRL) was conducted, such being a qualitative bibliometric analysis, with interpretative bias, which organizes and evaluates the literature based on domain, method and theory, many times in a manual form, with content analysis [50], such as in this article. This review was developed in five steps (Figure 1).

### 2.1. Bibliographic Search

The SRL first stage was conducted in March 2023, and constituted the search for scientific works in the Scopus platform, using the search string “*Chagas disease*” AND “*rural*”, delimiting to peer-reviewed articles, published at any given time. The articles found were exported to an electronic spreadsheet format, being listed in columns: author(s), title, year, publishing source, and DOI Scopus access link to the article’s abstract.

### 2.2. Selection of Articles by Criteria

In the second stage, a selection of articles by title has taken place, defining them as:

- (A) Adequate—discuss the occurrence and spread of CD, related or not to the presence and infection by *T. cruzi* in triatomines and/or animals;
- (PA) Possibly Adequate—might present variables which influence in the occurrence and spread of CD, related or not to the presence and infection by *T. cruzi* in triatomines and/or animals;
- (I) Inadequate—discuss the treatment and/or the physiobiological and clinical consequences of CD; they study the ecology of triatomines or their behavior in labs; they dissert about other diseases;
- (D) Duplicate articles.

In the third stage, we reviewed the abstracts of articles identified as adequate or possibly adequate in the second stage. This process involved updating the chart started in stage 1 with additional details, including the country of study, study area (rural, periurban, urban or nd—nondefined), objectives, methodology, results and conclusions/final considerations. Articles were then classified based on specific eligibility criteria:

- Inclusion criteria: (1) CD seroprevalence in humans, (2) infection by *T. cruzi* in triatomines, (3) infection by *T. cruzi* in animals;
- Exclusion criteria: (4) regarded other diseases; neglected tropical diseases in general; diagnostics and clinical treatments of CD; laboratorial studies; genotyping and/or ecology of triatomines without contribution for the analysis of risk variables for CD; diagnoses methodologies for CD; academic biographies; perception and intervention works; cases of individuals inhabiting only in urban area; (5) duplicated articles; and (6) without access to the article or abstract.

In the fourth stage, two exclusion criteria are added: (6) lack of full access or articles not in article format (e.g., reviews, dispatches, preliminary notes, or reports); (7) studies that do not report human CD seroprevalence, lack discussion on influencing variables for *T. cruzi* infection in humans, analyze *T. cruzi* infection in triatomines or animals, or focus solely on clinical diagnostics of CD.

Accepted articles included those that developed case studies with descriptive and/or statistical analysis. These studies covered various settings such as family groups [7], schools [32], communities [51], and involving blood donors [52] and/or patients of healthcare facilities [31]. Additionally, articles conducting statistical analysis using secondary data from individuals with confirmed CD serology, obtained from health systems or institutions, were also considered [53]. In the data spreadsheet collected during stage 3, information regarding the following were added: target audience/location, primary or secondary data, use or not of statistical analyses, predefined variables, and variable analysis.

### 2.3. Data Analysis

Initially, articles were grouped by study area (rural, urban, and/or periurban) and by the country where the research was conducted, creating a graphical representation to understand the geographical reach of CD and related studies. Subsequently, variables were identified and organized into specific groups based on their nature. These were categorized as independent predictor variables, which relate to socioeconomic, environmental, and cultural risk factors affecting individuals, whether healthy or contaminated, and may contribute to CD spread. Dependent predictor variables included epidemiological factors related to infected individuals and spatial dimensions that support CD spread. The variables identified were analyzed using a word cloud software to highlight frequently used terms in the articles. Variables, not always predefined in article methodologies, were discussed in relation to the realities of each individual or community studied, derived from the epidemiological profiles of the subjects. This included social, economic, cultural, and clinical characteristics, as well as environmental aspects within intra-, peri-, and extradomicile areas.

A graph was constructed with the percentages of articles that showed significance for CD. Additionally, forest plot graphs were constructed considering only the articles that presented the *p*-value of the analysis. The graph included the minimum value, the median, and the maximum value.

## 3. Results and Discussion

**Figure 2:**
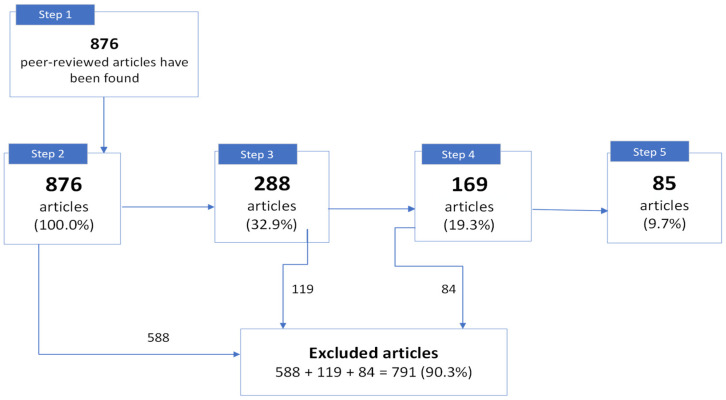
Flowchart of the amount of articles selected by stage.

We have identified 876 peer-reviewed articles published between 1953 and 2023, of which 85 articles have been selected for analysis and discussion (Figure 2).

The articles chosen for analysis were published between 1977 and 2022, and have presented spatial distribution of cases of CD, especially in the Americas. The studies were conducted in Brazil (24.7%), Argentina (22.4%), Mexico (16.5%), Venezuela (12.9%), Colombia (5.9%), Peru (3.5%), and Spain, Chile, and Ecuador (2.5% each). Additionally, Bolivia, Guatemala, Nicaragua, Panama, Paraguay, and Uruguay each contributed (1.2%). However, within this universe, the search and presence of the vector has been seen in 28.2% of the articles (24/85), in seven countries: Argentina, Brazil, Chile, Mexico, Panama, Peru, and Venezuela, as posteriorly observed in the item of the housing variable referring to the presence of triatomines in households.

Among the studies, 64.7% have been developed in rural areas. However, nearly 35% of the studies have also been conducted in urban and periurban areas, including urban and rural (29.4%); urban, periurban, and rural (3.5%); and periurban and rural (2.4%) areas. Despite rural areas remining a cause for concern due to the critical social conditions of certain regions associated with poverty, leading to precarious living conditions [54], the urbanization of CD has increasingly become a subject of study for similar reasons. This phenomenon triggers migratory movements in pursuit of better living conditions [55,56,57], thus drawing attention to two modes of transmission: through blood and congenitally. For example, between Red Cross blood donors, in the USA, infected individuals have been identified. These individuals have resided in precarious households and/or rural areas, and despite living in urban areas, performed nocturnal leisure or work activities in forested areas of the regions with documented reports of the existence of triatomines and animals hosts for *T. cruzi* [20], thereby increasing the likelihood of contact with triatomines. This is due to the fact that triatomines are wild or domiciled insects with nocturnal habits [7].

**Figure 3:**
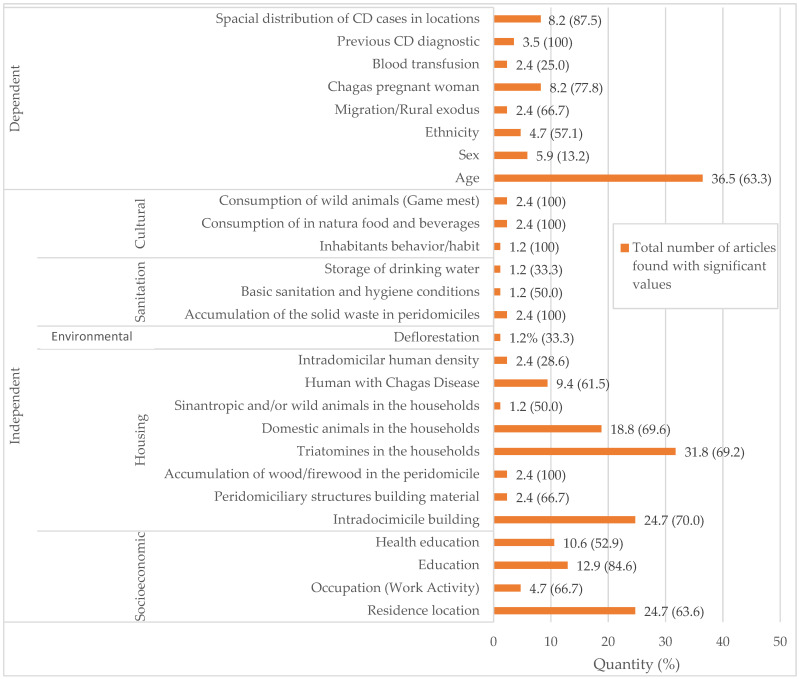
Dependent and independent variables found in the analyzed articles along with the quantity of significant results. Note: Dependent and independent variables found in the analyzed articles, presenting the number of studies with significant results for each variable in relation to the total number of articles analyzed (85 articles). The number of significant results in relation to the total number of articles for each variable is shown in parentheses.

After analyzing the articles, 27 variables were identified, comprising 19 independent predictors and 8 dependent predictors for CD spread (Figure 3). These variables were grouped based on prediction and dependence, mainly focusing on potential improvement actions, particularly in rural areas.

The independent variable predictors, categorized based on their influence on the environment and individual behavior, include socioeconomic and educational factors, housing conditions, environmental aspects, sanitation, and cultural factors. Dependent predictor variables are epidemiological factors related to individuals and the urban–rural divide, conditioned by individual seropositivity to facilitate the spread of CD. These variables were identified in multiple analyzed articles, suggesting that without the presence of chagasic individuals, they would not pose a risk for *T. cruzi* spread.

Figure 3 shows the number of articles with significant value in which variables were studied, relative to the total analyzed (59.5% 195/328). The median was calculated based on the *p*-values corresponding to the independent and dependent variables described in the studied articles. Significant results of independent variables appeared in 66.0% (134/203), with a median of 66.7%, and dependent variables in 48.8% (61/125), with a median of 65.0% The median allows observing that 50% of the results within the dependent and independent variables, respectively, presented significant results in 66.7% and 48.8% of the surveyed studies. Among the dependent variables, Age had with the highest number of studies and in 63.3% of significant results, while the variable Sex, and four other independent variables (Blood transfusion, Storage of drinking water, Intradomiciliar human density, and Deforestation), were below 50% of studies with significant results. Nonetheless, they can still provide evidence regarding their relationship to CD. Variables that had few studies or with statistical analysis 100% become limited in evaluating the condition.

The independent variables, such as residence location and education, intradomicile building, and presence of triatomines and domestic animals in the households, reflect the structural conditions of the intradomiciles. These factors remain in evidence in the discussions about CD spread, once that the precariousness house of building materials is a variable considered since the discovery of CD [42]. It is an aggravating factor for the proliferation of this anthropozoonosis’ vector [45,49].

**Figure 4:**
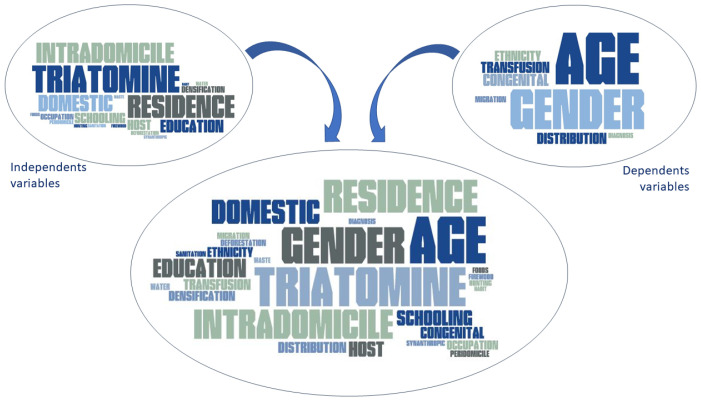
Word clouds presenting the intensity of appearance of the predictor variables in the analyzed articles.

It was perceivable in the word clouds (Figure 4) that other socioeconomic and housing variables linked to the socioeconomic vulnerability reflect the environmental context of CD transmission [26,45,58]. The studies also discuss variables such as the presence of triatomines, location of residence, domestic animal presence, health and education levels, and the presence of individuals with CD in households.

Between the dependent variables, just like when all variables are considered in the construction of the word clouds, age is highlighted because CD is a silent disease, which usually manifests in its acute state, in an average of 30 years after contamination [5].

**Figure 5:**
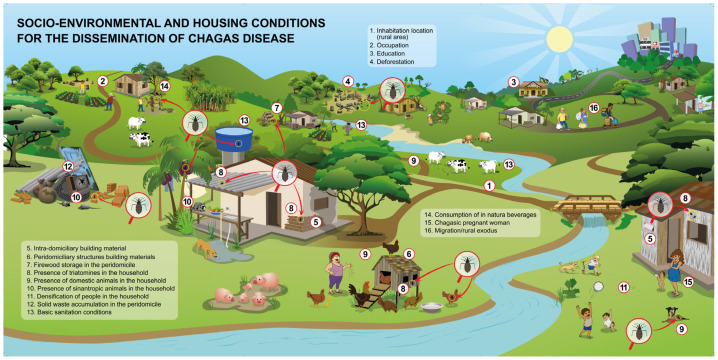
Presentation of certain variables within the rural context.

The analysis of each variable has revealed social vulnerability and its importance within the socio-environmental context of CD-hosting individuals, as found in the research of the analyzed articles (Figure 5). These socially vulnerable situations are attributed to household characteristics (availability of shelter, presence of cardboard or rammed earth roofs, dirt roof tiles, construction time, and domestic area) and socio-economic and demographic factors (overcrowding, area equivalency index, number of welfare payments received, number of wage earners, and education level) [51].

### 3.1. Independent Predictor Variables

**Figure 6:**
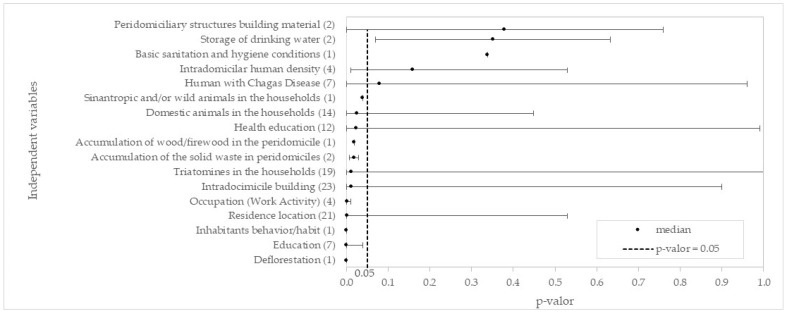
Forest plot for independent variables, using the *p*-values found in the researched articles, highlighting the minimum, maximum, and median *p*-values. Note: On the *y*-axis, in parentheses in front of each variable, is the quantity of *p*-values found.

Figure 6 (Forest plot) displays the range of *p*-values found in the articles examined in this study. Certain variables had limited reported values, and “Consumption of in natura food and beverages” and “Consumption of wild animals (Game meat)” are not shown due to the absence of reported *p*-values. Hence, variables with limited or absent *p*-values may represent areas for future research. Most variables exhibited a median *p*-value below 0.05. The few variables that exceeded 0.05 had limited *p*-values analyzed.

#### 3.1.1. Socioeconomic Variable

➢ RESIDENCE LOCATION

The residence location is related to the place where the household the individual lives at is built, be it in rural, periurban, or urban zone, community, municipality, or region. The geographic locations of households according to the definition of rural zones [59,60,61,62], periurban and urban [55,57,63], as well as vulnerability of the areas [29,64,65].

The rural household is still recognized by the WHO [3] as favorable for CD, by being a place where the way of life and coexistence with the natural environment is more propitious for vector and human contact, thus being considered an important characteristic in the epidemiologic analysis of a chagasic patient [26,66].

Discussions surrounding rural households delve into the various challenges posed by the realities faced by rural populations, which can impact the spread of CD. These challenges include low levels of education due to the remote nature of rural areas and limited access to public services [67], encompassing not only educational resources but also healthcare [56]. Additionally, there is the potential for engaging in less skilled work activities in wooded areas [68], which could lead to seasonal or permanent migrations [56,69]. The construction of precarious housing, often due to difficulties accessing suitable building materials [31,52], occurs alongside wild habitats of triatomines, facilitating vector domiciliation [62]. Furthermore, rural communities often coexist with synanthropic animals in and around their homes, serving as potential reservoirs for *T. cruzi* [64,70].

Overall, among the 32 articles with significant values for the residence location, 37.5% showed *p* > 0.05 or reported, without demonstrating values, that this variable does not significantly influence the number of seropositive cases of CD. Of these articles, only three provided the value, with *p* = 0.08, *p* = 0.529, *p* = 0.47. The majority, 62.5%, had *p* < 0.05, as evidenced in 18 rural communities in the state of Piauí, Brazil, due to housing improvements resulting from intervention programs [29], and between two Brazilian municipalities, justified by reports of coexistence with triatomines in households [65]. Among the five articles that presented the OR value, 80.0% found an increase from 1.1 to 7.46 times in the odds of finding an individual with CD due to the residence location. The greater chance of *T. cruzi* infection (7.4 times) was with coffee tree regions and significant climatic variation in the western part of Sucre, Venezuela [64]. The likelihood of acquiring a *T. cruzi* infection can vary significantly. In a study with puerperal women and their newborns in Venezuela, a 2.5-fold increase in the likelihood of acquiring a *T. cruzi* infection was observed among those residing in rural areas compared to those in urban areas [71]. This finding differs from research in Argentina, where the prevalence of CD in urban indigenous communities was 6.4 times higher than in rural communities [55]. The authors suggest that the recent rural exodus among indigenous populations involves individuals who previously lived in precarious socio-sanitary conditions.

➢ OCCUPATION (WORK ACTIVITY)

The occupation variable considered individuals which perform or performed nonqualified work, mainly rural area characteristic activities.

A transversal descriptive study conducted with CD patient data from regions of low, medium, and high risk for infection in Argentina, in their majority being residents of rural areas, found out that 88.57% performed nonqualified jobs [56]. Among these forms of work, it is the family agriculture/farming activity [2], which modifies the use of soil and is developed close to the triatomines’ natural environment, being found in Veracruz, Mexico, 3.8 times higher infection chance among farmer donors in relation to nonfarmers (*p* = 0.000) [52]. Another type of observed nonqualified job is the housewife occupation, which in both studied settlements had strong significance (Teotepec *p* = 0.010, El Jobo *p* = 0.022), once that they remained in households with high domiciliary infestation rate (39.3% Teotepec, 77.2% El Jobo) and lived with domestic animals infected by *T. cruzi* [72].

It is necessary to report another form of work, nonqualified and of subsistence, mentioned in studies conducted in the Brazilian Amazon, which presents risks, fitting the modality of extra domiciliary infection mentioned in the CD Epidemiologic Reported emitted by the Ministry of Health in 2021 [36]: extractivism, exemplified by the acai berry and piassava. Those are products originated from palm trees, habitat known as a natural wild triatomine environment [38,73], being collected and processed by individuals with an average age range of 30 years old, majorly of the male sex, and with physical ability to climb the acai tree to gather the berries [68], or collect piassava straws, being exposed to those insect bites [74].

➢ EDUCATION

In a study regarding education and social vulnerability, education has been presented as one of the conditioning factors which measure an individual’s vulnerability, especially in a context of poverty, as it gives them support in being acknowledged as a citizen and the understanding of their rights, duties, and benefits [58]. It was observed an inversely proportional relationship with seropositivity for *T. cruzi*, that is, the lower the education level, the greater the number of infected individuals [59,75].

For the Education variable, among the 13 articles with significant values, 15.4% showed values indicating that this variable does not significantly influence the number of seropositive cases of CD. The majority, 84.6%, had *p* < 0.05, being represented by illiteracy [76] and low education levels [53], both in the Brazilian Northeast, as well as for situations of low education levels among women found in the municipality of Pore, in Casanare, Colombia [31]. Low education levels (illiteracy to incomplete high school) are associated with the marginalization of the population and poverty [77,78], to social vulnerability [30,62,63], and to less qualified jobs [56]. It is noteworthy that congenital transmission cases in Cumural, Meta, Colombia, among which greater seroprevalence in children of mothers with below-secondary education levels have been found [67].

➢ HEALTH EDUCATION

Education is linked to a population’s “medical culture”, impacting their ability to absorb information and practice self-care regarding health [79]. Basic education can facilitate hygiene practices such as handling acai berries to prevent oral transmission [66]. For instance, in Porto Letícia, São Paulo, Brazil, the lack of knowledge about CD and its vector hinders community support for intervention programs, as entomological surveillance relies on the population identifying and reporting insects. This lack of awareness extends to school-age children, indicating an education gap and hindering intervention efforts [80].

The studies were based on knowledge of CD and its vector, the triatomine [9,59,78,80,81,82,83]. Analyzing health education overall, among the 17 articles with significant values found, 47.1% showed *p* > 0.05 or reported, without demonstrating values, that this variable does not significantly influence the number of seropositive cases of CD. Of these articles, only five provided the value, with *p* = 0.49, *p* = 0.39, *p* = 0.99, *p* = 0.056, and *p* = 0.31. The majority, 52.9%, had *p* < 0.05, among which the knowledge about triatomines among *T. cruzi* positive patients, monitored at the Universidade Estadual Paulista, Brazil, especially those born before 1983 [84]. Similarly, among blood donors in Veracruz, Mexico, merely seeing or recognizing the triatomine has doubled the odds of seropositivity [52]. The two articles that presented the OR value found an increase from 1.69 to 3.26 times in the odds of reducing CD due to health education.

#### 3.1.2. Housing Variable

➢ INTRADOMICILE BUILDING

In the literature, intradomicile building materials are widely recognized as influential factors in the spread of CD. Structures built with precarious materials create a conducive environment for insects, providing shelter and facilitating their proliferation [27,52,61,65,85,86,87,88,89,90,91,92,93,94,95]. The research also found infected individuals living in well-structured homes, but who had previously lived in households with precarious conditions, like Chagas-infected pregnant women from Pore, Casanare, Colombia [31]. Improving household structural conditions is seen as a protective measure due to the recognized risk factor of precariousness. A study in Campinas do Piauí, Piauí, Brazil, observed a reduction in cases after an intervention program [29].

Overall, among the 29 articles with significant values for Intradomicile building, 31.3% showed *p* > 0.05 or reported, without demonstrating values, that this variable does not significantly influence the number of seropositive cases of CD. Of these articles, only five provided the value, ranging from 0.04 to 0.90. The majority, 68.7%, had *p* < 0.05, highlighting the building material, particularly the lack of plaster [72,96]. The two articles that presented the OR value found an increase from 2.1 to 2.33 times in the odds of finding an individual with CD due to Intradomicile building. One can take as an example that the type of building material used (plant matter, adobe, or unfinished brick walls) can increase the chances of infection by 1.7 times [97].

➢ PERIDOMICILIARY STRUCTURE BUILDING MATERIAL

The precarious conditions, like cracked walls and a microclimate conducive to the lifestyle of kissing bugs, extend beyond the confines of households. The infrastructure of peridomiciles can also provide refuge for triatomines, serving both to protect households and as a connecting pathway between natural habitats and homes [64,98].

In the peridomiciles, especially in rural households, besides animal nurseries, toolsheds and production storages are also built in them, identified, in rural Venezuelan communities in the state of Lara, as protection factors [82]. On the other hand, wild animal burrows and bahareque annex buildings (*p* < 0.05) found by the same authors have been considered as risk factors for allowing the sheltering of *T. cruzi* host animals and insects, respectively [82]. Additionally, various materials stacked in yards, including wood, firewood, and waste, among others, were discovered in households within rural communities in Sucre, Venezuela [64].

➢ ACCUMULATION OF WOOD/FIREWOOD IN THE PERIDOMICILE

The accumulation of wood/firewood in the peridomicile has been considered as a predictor variable since wood is a natural product, with similar characteristics to the wild habitat of the thumbtacks, attracting them, such as wattle and daub used in the construction of intradomiciles [99]. As an example, it was found that the odds of *T. cruzi* infection were 2.1 times higher in Sucre (Venezuela) due to the presence of firewood [64]. When both firewood and waste were present, the odds were 2.5 times higher in rural communities in the coastal provinces of Ecuador [91].

➢ TRIATOMINES IN THE HOUSEHOLDS

The presence of triatomines in the wild and domiciliary environments, when accessed, make exposure possible and increase the odds of contact between vector and human beings. This variable considered the presence of the vector insect of American trypanosomiasis in the domiciliary environment, intra- or peridomicile, as well as forms of exposure/contact with triatomines: seeing it in the house, having contact with the insect, or being bitten by it [27,65,81]. Once infected, the feces of these insects may also contaminate food and even water which will be drank by inhabitants [8].

Despite the vector transmission interruption achieved by 18 American countries, either at the national level or part of their territories [35], the presence of triatomines in households continues to be a predictive factor. This is because these hematophagous insects have nocturnal habits [6], turning both inhabitants and domestic animals into sources of nourishment. This scenario makes infection possible through a human or animal host, facilitating the spread of *T. cruzi* through their free roaming and feeding [93,95,100,101,102,103,104].

Among the 37 articles with significant values found for triatomines in the households, 32.4% showed *p* > 0.05 or reported, without demonstrating values, that this variable does not significantly influence the number of seropositive cases of CD. Of these articles, only five provided the value, ranging from 0.09 to 1.00. The majority, 67.6%, had *p* < 0.05, showing it to be an important variable, as exemplified by a study in Amamá community which found that *T. cruzi* incidence increased in children under 16, with domestic T. infestans abundance rising from 1 (1988) to 11 (1992) insects per person-hour (*p* < 0.001). Triatomines were also found feeding on human and animal blood in re-infested houses of infected children (12.1%), without travel or transfusion history, suggesting high human–insect contact [105]. The seven articles that presented the OR value found an increase from 1.9 to 11.7 times in the odds of finding an individual with CD due to triatomines in the households. This scenario can be illustrated by the 1.9 times increase in human infection odds when considering the density of infected vectors [106].

The vulnerability of the population to the invasion of bugs into the domestic environment is observed due to the proximity of dwellings to palm trees in the wild habitat [38]. For example, the predominance of the species *Rhodnius pallescens* is related to the abundance of *A. butyracea* palms in some rural communities of Santa Fé, Panama, as well as the presence of another species, *Triatoma dimidiata* [107], the second most important vector for CD in Panama, although with wide distribution and low density [108]. In contrast to the high infestation of *T. didimiata*, a low human seroprevalence (2.3%) found in the rural community of San Juan Batista Sakcabchen, located in the Yucatán peninsula, Mexico, was observed. Here, natural triatomine infection was observed as a risk factor. However, it is important to consider the characteristic of this specimen as a “poor vector” due to its long defecation time after feeding [109].

In studies where no search for triatomines was conducted in the houses of infected individuals, but rather only questioning about the presence of these insects in the households [89,95], the influence of the kissing bug in the domestic environment has been observed. This is supported by the identification of a two-fold increase in the odds of *T. cruzi* infection among young people aged between 10 and 20 years, confirming the presence of vectors in their households, in rural communities in Argentina (OR = 2.08) [110].

Positive associations have also been observed in other studies, such as with chagasic pregnant women from Casanare, Colombia, where it was identified that they had been in contact with the insect at least once [59]. In this contact, they may have been bitten by the bug, as well as chagasic women and children from the Sudzal and Teya communities, rural Mayan villages in Yucatan, Mexico [83], corroborating with other studies [9,19,111].

Concluding this variable, exposure and contact with triatomines are not confined to domestic settings, as noted by the Brazilian Ministry of Health [36]. Researchers observed triatomine attacks on workers during piassava gathering in the Brazilian Amazon region, indicating a link between *T. cruzi* seroprevalence and human exposure to the vector in a wild extradomiciliary environment [74].

➢ DOMESTIC ANIMALS IN THE HOUSEHOLDS

Once the movement of domestic animals between environments may cause both the passive transportation of triatomines in their fur and the increase of infection odds in these animals, their coexistence in domestic environment becomes a predictor factor for human infection [31,59,106]. A relative risk of 1.7 was found in relation to the free roaming of animals around the house and human seropositivity in rural locations of Sierra Mixteca, Mexico [96].

The presence of *T. cruzi* host animals increases the likelihood of disease transmission through the domiciliary vector cycle, as triatomine bugs are hematophagous and inhabit environments where infected and noninfected individuals coexist. This situation can lead to multiple feeding sources in a single night, facilitating the spread of the protozoan [90,93,105]. With emphasis on dogs, the domestic animals usually roam freely in every domicile strata. However, the presence of animals napping in the intradomicile may both attract triatomines as well as being a predictor factor for the human infection by *T. cruzi*, once the triatomines have more food sources available in that household [64,82,103].

Overall, among the 20 articles with significant values for domestic animals in the households, 35.0% showed *p* > 0.05 or reported, without demonstrating values, that this variable does not significantly influence the number of seropositive cases of CD. Of these articles, only three provided the value, ranging from 0.15 to 0.449. The majority, 65.0%, had *p* < 0.05. For example, in northwestern Argentina rural communities, there is a strong association between child infection and the number of infected dogs [86]. The five articles that presented the OR value found an increase from 1.9 to 11.0 times in the odds of finding an individual with CD due to domestic animals in the households. Dogs and cats are the most frequently mentioned animals as hosts. For example, increases of up to 9 times in the odds of CD have been reported in rural communities in Argentina [106], and 6 times more chance in children under 16 years old in indigenous and creole communities in Argentina [40].

➢ SINANTROPIC AND/OR WILD ANIMALS IN THE HOUSEHOLDS

Wild mammals, especially marsupials, are recognized as predictors of *T. cruzi* infection, as they serve as potential hosts for the protozoan and have unrestricted access to households’ peri- and extradomiciles [38]. The presence of these animals has been linked to family outbreaks of oral transmission, as they have been observed wandering in areas where fruits are stored for consumption, which could lead to food contamination through their anal glands [112].

In a comparative study between chagasic individual without cardiac insufficiency treated in rural health clinics in the state of Lara, Venezuela, a significant association of CD with the presence of wild reservoirs in the household has been found. This association resulted in an increase of 2.2 times in the infection odds (OR: 2.27, *p* = 0.04) [81]. On the other hand, no CD association with the presence of sinantropic animals has been found (*p* > 0.05) in Venezuelan rural households in Sucre, even when finding piled waste in their peridomiciles [64], a fact which makes the environment propitious for the roaming of sinantropic animals and disease vectors, such as rodents [113,114].

➢ HUMANS WITH CHAGAS DISEASE

It is worth observing that, in studies in which separated maternal infection has been verified, such has been considered as another variable (dependent), facing the possibility of congenital transmission, reflecting the coexistence of family with CD, with or without cohabitation [31,60,64,115].

Among the 11 articles with significant values for human with CD, 45.5% showed *p* > 0.05 or reported, without demonstrating values, that this variable does not significantly influence the number of seropositive cases of CD. Of these articles, only four provided the value, ranging from 0.08 to 0.96. The majority, 54.5%, had *p* < 0.05, as illustrated by cases such as the epidemiological profile of chagasic pregnant Latin American residents [116], and likewise with chagasic immigrants residing in Palma and Son Pisa [27], both in Spain. The two articles that presented the OR value found an increase from 1.75 to 4.8 times in the odds of finding an individual with CD due to humans with CD. The fact of patients having coexisted with host family members at any given time, without cohabitation, finding an increase of 1.7 in the odds of seropositivity due to the aforementioned variable [71].

In rural communities of indigenous and creole individuals in Chaco Province, Argentina, each infected cohabitant has been linked to a 40% increase in individual human infection rates. This is particularly notable as seroprevalence was higher among inhabitants with prior exposure to *T. infestans* (79% were residents in infested houses [103]). Additionally, in Argentina, a descriptive analysis revealed that the presence of humans and infected triatomines sustains the domestic vector cycle [102]. This contrasts with a negative multivariate association found in children, where seroprevalence was not significantly associated with the abundance of infected *T. infestans* or the presence of an infected child (*p* = 0.81) [105].

➢ INTRADOMICILIAR HUMAN DENSITY

Once the triatomines can proliferate and spread throughout the household, arriving to the dorms and intradomiciles, it is understood that the degree of significance of the number of family members with child seropositivity (*p* < 0.01), as found in three hamlets in the interior of Goiás, Brasil [100]. This fact was also observed in individuals between 10 and 20 years old in rural communities of northwestern Argentina, in which was identified the seroprevalence association for *T. cruzi* with family overpopulation in their households (*p* = 0.02) [110].

In the articles, it was mentioned to the influence of the number of inhabitants in a household, the organization of the domiciliary environment, and the sleeping of inhabitants, forcing them to sleep against the walls, easing the contact with insects [26,79]. Likewise, it reinforces the relation of CD with social vulnerability, as the excessive densification is characteristic of housing deficit, once that the number of rooms in the intradomicile, save for the bathroom, is equal to the number of rooms serving as dormitories [117].

#### 3.1.3. Environmental Variable

➢ DEFORESTATION

Deforestation, attributed as a predictor for CD, arises from factors like extensive migration, urbanization, agricultural expansion, and livestock farming in rural regions [63]. The adjacency of homes to deforested zones can draw triatomines due to artificial illumination [118], leading them to seek refuge and food sources within households [62].

In study conducted in five administrative regions of the municipality of Barcarena, in the state of Pará, located in the Brazilian Amazon region, a significant association between location and ACD and deforestation cases has been established (*p* = 0.0001) [63]. By contrast, in Mexican rural communities, relation between the absence of human seropositivity and lack of forest and more humid soil has been found in Tierra Blanca, and among high seropositivity rates and the presence of perennial tropical forests in drier soils in Tezonapa (both, *p* = 0.002) [9]. Similar results were observed regarding changes in closed forests and cultivated vegetation, with the occurrence of at least one person infected by *T. cruzi* in the rural communities of the Province of Córdoba, Argentina (*p* > 0.05) [119].

#### 3.1.4. Sanitation Variable

➢ ACCUMULATION OF SOLID WASTE IN PERIDOMICILES

The accumulation of solid waste in peridomiciles has been identified as a predictor factor in CD, in the measure that it creates propitious conditions for sheltering and proliferation of vectors in household yards, besides attracting more sinantropic animals, knowns as *T. cruzi* potential hosts [64,82,114].

The burial and burning of waste have been verified as risk factors and are considered significant in the association of CD spread (respectively, *p* = 0.008 and *p* = 0.03) in Venezuelan rural communities [64]. However, despite being a polluting action, burning was identified also as a protection factor (OR = 0.52), for reducing the infection risk by eliminating the material piled up in peridomiciles; not finding the same characteristic for the burial (OR = 3.2), supposing that there was undisclosed information and the waste has not been entirely buried [64]. Likewise, in multivariate analysis, an increase of 2.5 times has been found in the chance of *T. cruzi* infection for inhabitants in households with waste and firewood accumulation in peridomiciles (POR = 2.51) in coastal regions of Ecuador; not finding the same association in households of the uplands region [91]. Furthermore, the accumulation of waste and organic matter outside of the households has been considered a protective factor against CD seropositivity [91].

➢ STORAGE OF DRINKING WATER

In a case study of chagasic outbreak conducted with a family group of the Yaguapita community, state of Miranda, Venezuela, it has been observed that patients did not present any inoculation chagoma, that is, no protozoan entry symptom. Thus, an epidemiologic study in the patients’ households has been conducted and, in dialogues with them, they have informed that they have found “chipos” (triatomines) in the kitchen, food prepping location, and in containers which were not frequently used [7]. Although the patients claimed to always wash food before consumption, the possibility of contaminated water consumption was considered due to storing it in an open pot near a window, without a protective screen, allowing direct contact with the outside environment and potential access for triatomines into the household [7]. An intriguing aspect of this study was the investigation into epidemiological factors contributing to *T. cruzi* infection in this family. Information regarding the family dog revealed that it exhibited the same symptoms as the patients two months prior to their diagnosis and subsequently passed away [7].

➢ BASIC SANITATION AND HYGIENE CONDITIONS

The absence of basic sanitation and hygiene conditions is a reality of many rural communities which can result of the extractivist and farming way of life, compatible to their income, housing conditions, and education level, characterizing their social vulnerability [68]. Another reality in other rural households is that, due to the improper disposition of manure and the absence of drinking water supplying systems, inhabitants need to move between peri- and extradomiciliary environments to defecate or gather water at springs, exposing themselves to the kissing bug’s natural environment [120].

Upon associating basic health and sanitation conditions with human seropositivity for *T. cruzi* in a Venezuelan indigenous rural community, situated in the National Park Serra de Perijá, odds of infection 3.2 times higher have been observed for individuals which had contact with soil contaminated with animal feces living in its peridomiciles [95]. Notwithstanding, in the same study, the authors did not find significant association (*p* > 0.05) of CD seroprevalence with the act of washing hands before eating and/or preparing food [95]. This finding corroborates the research conducted with pregnant women in the state of Guajanuto, Mexico, which considered other sanitation aspects, also not finding association with CD: drinking water supplying (OR = 0.8; *p* = 0.633) and drainage and sewer versus cesspool (OR = 0.5; *p* = 0.338) [61].

#### 3.1.5. Cultural Variable

➢ INHABITANT BEHAVIOR/HABIT

Cultural variables include habits and behaviors such as visiting relatives in potentially endemic areas, which increases exposure to the vector, and changing sleeping locations during peak seasons, when individuals may sleep on the household balcony [105]. According to the authors, sleeping in the intradomicile’s external area, with an infested household, reduces the possibility of kissing bug bites in that individual. Another risk behavior, stemming from the population’s culture, is nomadism [103]. A study conducted in indigenous and creole communities in Argentina revealed that indigenous nomadism doubles the chances of infection compared to creoles, as their mobility increases the likelihood of occupying houses infested with triatomines [103].

➢ CONSUMPTION OF IN NATURA FOOD AND BEVERAGES

The habit of consuming *in natura* foods and beverages is a predictor action of CD oral transmission, being that approximately 20 ACD outbreaks have been registered in the center-north and west of Venezuela in the last two decades [121]. In Brazil, oral transmission was identified less than 20 years ago, with a higher incidence in the Amazon Region, according to the Epidemiological Report of the Ministry of Health [36].

A survey of confirmed CD cases registered by SINAN, between 2009 and 2016, in Acre, a Brazilian state, identified oral transmission through *in natura* beverages as the most common contagion form (76.2%), with a greater number of cases in the municipality of Feijó, which has a big acai production [68]. The pulp of this fruit is culturally eaten *in natura* in the region or frozen and exported to other states [13]. Due to the ACD outbreaks stemming from oral transmission in Brazil, the Ministry of Agriculture and Supplying, through Normative Instruction n. 1, from January 7th, 2000, approved the technical regulation for the fixation of Identity and Quality standards for fruit pulp, among which the acai berry pulp is listed [122].

Other cases with positive association with CD were reported with consumption of homemade guava juice *p* < 0.05 [32] and with infected triatomines and sinantropic animals near the places where ripe fruits were stored, suggesting oral transmission [112].

➢ CONSUMPTION OF WILD ANIMALS (GAME MEAT)

Another predictor action of oral transmission is the consumption of meat or blood of wild animals, known as game animals, the oldest case reported having occurred in 1936 in the Argentinean Chaco, of a sick child, due to the ingestion of a beverage suggested by healer containing herbs and armadillo blood [123].

In a study conducted with patients of the National Infectology Institute Evandro Chagas (INI), Oswaldo Cruz Foundation (FIOCRUZ), between 1986 and 2011, and farms from the north rural zone of Rio de Janeiro, Brazil, the inquiry to seropositive individuals and the discovery of infected wild animals suggested the consumption of game meat as a source of oral transmission [70]. There may still be an aggravating factor, such as that reported by settlers of São Paulo, in Brazil, which informed having the habit of manipulating and consuming undercooked meat of armadillos, spotted cavy, and foxes, among other mammals acquired from hunting [80].

### 3.2. Dependent Predictor Variables

**Figure 7:**
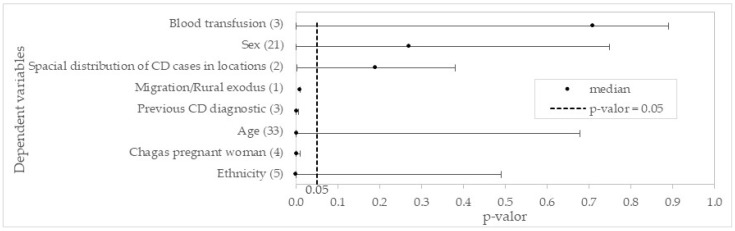
Forest plot for dependent variables, using the *p*-values found in the researched articles, highlighting the minimum, maximum, and median *p*-values. Note: On the *y*-axis, in parentheses in front of each variable, is the quantity of *p*-values found.

Figure 7 presents the variation of the *p*-value found in the articles surveyed in this research. Some variables had few values reported, so these variables may represent gaps for future research. The median of the *p*-values found in the articles highlights that the majority of variables are significant and can be used as predictors.

CD is a disease that, upon manifesting itself, usually attacks people of more advanced ages, when the disease has already moved from acute to chronic stage, not presenting a direct relationship to sex or ethnicity with the acquisition of the infection. Previous CD clinic diagnostics and prenatal exams serve as forms of awareness, with the risk of spread conditioned upon confirmation of *T. cruzi* seropositivity, such as through blood transfusion. Similarly, the migration or rural exodus of infected individuals is recognized as a significant factor in the spread of CD, particularly when it involves movement to nonendemic countries. Lastly, the spatial distribution of CD cases might generate risk by proximity and coexistence of infected individuals, when there are propitious environmental conditioners.

➢ AGE

The variable age has been found in studies developed in Brazil, Argentina, Bolivia, Colombia, Spain, Ecuador, Mexico, Peru, and Venezuela. For being a silent disease that reveals its symptoms only in 10 to 30 years after the protozoan infection [3], in most research, age is found in a directly proportional relation to the CD diagnosis, that is, older people have more probability of discovering the infection [31,53,101,115,124,125,126,127,128,129,130].

Overall, among the 48 articles with significant values for age, 37.5% showed *p* > 0.05 or reported, without demonstrating values, that this variable does not significantly influence the number of seropositive cases of CD. Of these articles, only four provided the value, ranging from 0.363 to 0.678. The majority, 62.5%, had *p* < 0.05, with communities in São João do Piauí, Brazil, serving as an example, where a significant association of seropositivity within the age range over 60 years old has been identified (*p* < 0.001) [2]. Still in Brazil, state of Minas Gerais, the average age for *T. cruzi* seropositive individuals was 56.19 years old, with a significant association of *p* < 0.001 [65]. Similarly, in rural communities of the Argentinean Chaco, the seroprevalence increased significantly with age (*p* < 0.001) [103]. The six articles that presented the OR value found an increase from 1.018 to 9.2 times in the odds of finding an individual with CD due to age. In rural communities of Piauí, Brazil, the parasitic infection was 4.4 times greater in the population aged from 0 to 10 than 11 to 60 years old (*p* < 0.001) [29].

Some articles also found minors aged below 10 years old were infected [32,86,87,91,106,131], suggesting the possibility of active vector transmission or congenital transmission, passed down from mother to child, as was found in rural Peruvian communities, where the prevalence of *T. cruzi* in kids aged 15 and below was 17.2% (IC 95%: 9.6–24.7%) [132] and 16.7% among children of indigenous and creole communities of the Argentinean Chaco (*p* = 0.01) [103].

➢ SEX

This variable was present in 43.5% (38/85) of the articles analyzed, which verified as significant or not the difference between male and female for chagasic seropositivity [76,133,134,135,136]. However, despite men presenting more contact possibility with triatomines due to their work activities in the thumbtack’s natural environment [13], and being majority among cases of trypanosomiasis [137], among the articles which consider “sex” as significant variable for CD spread (5.9%—5/85), all of them present a greater number of women infected [29,101,138,139]. It is believed that this occurred because, in rural areas, women tend to spend more time in households engaged in domestic activities, thereby increasing their exposure to triatomines while residing in infested areas [72].

Among the 36 articles with significant values for sex, 91.7% showed *p* > 0.05 or reported, without demonstrating values, that this variable does not significantly influence the number of seropositive cases of CD. Of these articles, only four provided the value, ranging from 0.06 to 0.749. The minority, 8.3%, had *p* < 0.05. In this context, there may be contrasts within the same region, as seen in rural communities in Guatemala, where it was identified that 50.6% of the seropositive were women, not finding significant difference in the location of San Miguel Huite (*p* = 0.28), but a quite significant one in the location of Primera Sabana (*p* = 0.01) [140]. Only one article presented the OR value, finding an increase of 1.9 times in the odds of finding an individual with CD due to sex [115].

➢ ETHNICITY

Studies have been developed in Brazil and Argentina, with the individuals’ self-declaration as black, brown, indigenous, and white, among others, being observed.

Upon identifying the epidemiologic profile of the seropositive individuals from the Brazilian municipalities of Abaetetuba and Barcarnena, state of Pará, were identified, respectively, brown majority (*p* < 0.0001) [63] and mixed majority (*p* < 0.0001) [62], the authors mention the CD social characteristic of individuals in social vulnerability situation [62,63]. In the Argentinean Gran Chaco, in a study in indigenous and quilombola communities, greater infection odds have been identified regarding indigenous people (*p* = 0.002) due to the nomadic culture and greater possibility of inhabiting infested houses, also, within a social vulnerability context [103]. This fact reinforces the statistical difference found between the seropositivity in indigenous and creole communities (*p* = 5 × 10^−4^) in the same region, observing an increase in seroprevalence in locations more distanced from the urban centers and the difficulty of access to said communities, even for intervention programs, due to their isolation [141].

➢ CHAGASIC PREGNANT WOMAN

This variable referred to the possibility of *T. cruzi* protozoan transmission from mother to child, also known as vertical or congenital transmission. In Latin America, nine thousand new cases of congenital transmission are reported [35]. This variable has been deemed as a predictor in research in rural and urban places [82,84,142,143], as well as children and immigrants.

In an inquiry conducted with Bolivian immigrants registered with general practitioner doctors in Mallorca, Spain, the record of chagasic mothers has been identified in 50.7% of the seropositive individuals and strong significance for *T. cruzi* spread [27].

Maternal seropositivity with infant seropositivity for CD was significant in indigenous and creole communities of Pampa del Indio, Argentina (*p* < 0.001) [103], as well as in rural communities in the state of Goiás, Brazil (*p* < 0.01) [100]. In rural areas of the eastern region of Paraguay, in a study involving Chagas-infected children, none of them had received blood transfusion, no vector had been found in their homes, and all were offspring of Chagas-infected mothers [144].

Studies identifying seropositivity in pregnant women and those of fertile age, with or without confirming congenital transmission, emphasize the importance of public health policies for diagnosis and treatment of mothers and children in both rural and urban settings [55,61]. Early treatment of newborns upon confirmed diagnosis can increase cure likelihood. Additionally, decisions on breastfeeding by Chagas-infected mothers and anti-parasite treatment for fertile-age women may reduce vertical transmission risk [5].

➢ BLOOD TRANSFUSION

For being a silent and asymptomatic disease in most hosts, there is the possibility of CD transmission in urban and nonendemic areas, stemming from blood transfusion [42]. The articles verify the records of blood transfusions received by seropositive individuals, as awareness of being a host may prevent the spread of the disease and increase the likelihood of treatment [97,145]. This condition was observed in rural communities of Piauí, Brazil (*p* = 0.0017), with a 1.7 times higher risk of contracting CD in relation to nontransfused seropositives (RR = 1.74) [75], as well as in Spain (*p* = 0.000), and in 9.3% of Latino-American pregnant host patients, leading them to treatment to prevent congenital transmission [116]. This investigation shows the importance of triage both of pregnant women and blood/organ donors. It is not without reason that Brazil has put among one of their mitigating measures against CD the verification of infection by *T. cruzi*, being recognized since 2006 not only by the eradication of *T. infestans* vector, but also by controlling the blood transmission through triage before blood transfusion [139].

➢ PREVIOUS CD DIAGNOSIS

The articles have been generated through epidemiological surveys applied to the sampled population, emphasizing the importance of this knowledge to prevent the spread of the disease and enable its treatment [81].

When comparing rural individuals with and without cardiac insufficiency (CI) assisted in hospitals of Venezuela, a greater seropositivity association of the participants has been found, with the age and knowledge regarding triatomines, being the previous clinic diagnostic of the disease (OR: 5.36) significant only for the individuals with CI, making it possible for them to have a better understanding of their symptoms’ causes [81].

In another context, with rural and urban populations of the Casanare department, in Colombia, a significant seropositivity association has been found in these individuals due to the fact that they have been through previous CD triage (*p* < 0.0001). The screening was conducted to alert the population about the possibility of the disease spread, once that they live, in their majority, in households with precarious building materials and close to the triatomines’ wild ecotope, mainly palm trees [97].

➢ MIGRATION/RURAL EXODUS

Migration presents risks both for the reappearance and the spread of CD, once that the infected hosts are humans [146]. Rural exodus, as well as the migratory movement between municipalities, states, and countries, has been modifying the CD epidemiologic profile, from the search of individuals for better work and life conditions [77,80,126]. This dynamic possibly leading to urbanization and increase of the epidemiologic geographical area of *T. cruzi* [131,147]. This variable comprised the articles which have presented it ipsis literis, or as “birth in endemic areas”.

The influence of this variable was significant in a study in Peru (*p* < 0.01) [145] and among settlers in Porto Letícia, state of São Paulo, Brazil [80]. Being the majority of the infected individuals from rural areas, it reinforces the studies about the relation of CD with these areas, which come since the discovery of the disease by Dr. Chagas, considering the rural area as endemic [42].

➢ SPATIAL DISTRIBUTION OF CD CASES IN LOCATIONS

The researches sought to analyze the spatial distribution of infected individuals in a specific study area, with the use of software tools, considering environmental conditions, habitability, and/or geographical location with individuals carrying CD. Using primary or secondary data, it is possible, through spatial distribution, to find a significant relationship with the number of cases of CD [9,62,63,119,131,148].

It is noteworthy that climate conditions, upon interfering in the dispersion of triatomines, may influence their movement between wild and domestic environments and domiciles, depending jointly on other factors, such as feeding, physiologic characteristics, and the insect’s life stage (nymph or adult) [149,150,151].

## 4. Final Considerations

In the several contexts encountered, urban, periurban, and especially rural, the social vulnerability, including poverty and low educational level, as well as the lack of information, lead the individual to: i) living in environments where inhabitability is lacking, ii) perform limited work activity; iii) developing habits and behaviors which condition them to an environmental insalubrity situation, among other conditions. This situation is conducive to the access of vectors and pathogens of anthropozoonoses such as CD.

The variables had their importance within each group, which generally proved significant in most situations. However, it is necessary to analyze the application of each variable punctually for a more accurate decision.

The search for CD variable predictors has presented vulnerability of the socioeconomic and basic sanitation conditions factors, observed the absence of health education, reflected in varied realities, within the housing and extra domiciliary context.

Low education level may lead an individual to a limited work condition, such as rural work activities, which entails low profit and social vulnerability, making them remain in rural areas or migrating to periurban areas in cities, with little access to public health services, sanitation, transport, education, and leisure, among others.

They build households with materials of precarious intradomiciliary structure, in which fissures appear and constitute microclimates, propitious for the proliferation of CD vectors. Furthermore, the building of nests in the peridomiciles for domestic animals, which roam freely between intra-, peri-, and extra domiciles, especially dogs and chickens. These animals, besides serving as passive transportation for thumbtacks, are potential *T. cruzi* hosts, being recognized as parts of the domestic vector cycle of the CD, once that they roam around the areas of food storage and nap within intradomiciles.

The households, for being built in more remote areas, in deforested areas close to the triatomine wild ecotopes, make it possible for these insects, with a flight capacity range up to 1 km [4], the occupation and sheltering in intra- and/or peridomicile, possibly infecting animals and humans or spreading *T. cruzi*, in case there is a host in said domicile environment. Facing such invasion, they may also contaminate food and/or water, which due to circumstances of lack of water treatment system, is stored in buckets, most likely unlidded, for human consumption. Within said context, besides the culture of in natura food consumption, which may be contaminated, the rural residents usually practice the accumulation of solid waste in peridomiciles, which serves as local shelter and proliferation of kissing bugs. However, the unfamiliarity regarding the vector, the CD, or contraction forms, amplify the possibility of proliferation of such anthropozoonosis, while observing the conditions of hygiene, sanitation, and individuals’ behaviors.

## 5. Conclusions

With the development of such work it was possible to conclude that the predictor variables for CD transmission may be determined by the presence of an infected individual and their influence on environmental conditions and behaviors, potentially facilitating disease spread. Despite the longstanding recognition of household structure as a relevant factor in CD, other variables have emerged as significant, reinforcing the socioeconomic and cultural dimensions of vulnerability to the disease. CD is not confined to rural areas, given its various transmission modes. Factors such as deforestation, housing construction near vegetation, and work in wild environments are crucial considerations, impacting the habitats of disease vectors and increasing human–vector contact. Rural communities’ isolation, limiting access to healthcare and education, complicates disease prevention, diagnosis, and treatment efforts, necessitating coordinated action by public institutions and at-risk populations to control vector populations and disease spread. Access to basic sanitation, healthcare, and health education is vital in preventing vector proliferation and waste accumulation in living spaces. Epidemiological variables highlight the diverse detection methods and spatial distribution of CD. Ultimately, a combination of health education and intervention programs, supported by community involvement, is key to controlling vector populations, conducting entomological surveillance, and implementing sanitary measures.
